# Understanding Novel Metaphors: A Milestone in the Developmental Trajectory of Children with Agenesis of the Corpus Callosum?

**DOI:** 10.3390/brainsci10100753

**Published:** 2020-10-19

**Authors:** Sergio Melogno, Maria Antonietta Pinto, Chiara Pollice, Fausto Badolato, Guido Trasimeni, Pasquale Parisi

**Affiliations:** 1 Department of Developmental and Social Psychology, Faculty of Medicine and Psychology, “Sapienza” University of Rome, 00185 Rome, Italy; mariantonietta.pinto@uniroma1.it; 2 Faculty of Psychology, “Niccolò Cusano” University of Rome, 00166 Rome, Italy; chiara.pollice@unicusano.it; 3 NESMOS Department, Faculty of Medicine and Psychology, “Sapienza” University of Rome, 00189 Rome, Italy; faustobadolato23@gmail.com (F.B.); guido.trasimeni@uniroma1.it (G.T.); pasquale.parisi@uniroma1.it (P.P.)

**Keywords:** agenesis of the corpus callosum, children, novel metaphor comprehension

## Abstract

This study explores novel metaphor comprehension in a 7.2-year-old child (conventionally called RJ) with complete and isolated agenesis of the corpus callosum (ACC). RJ’s cognitive level was adequate for his age as well as most of his linguistic competencies. The child’s performance was compared to typically developing (TD) controls on a test assessing novel metaphor comprehension for preschoolers. RJ’s performance showed a delay of about three years in relation to the expected level for his age, and also a significant gap compared to the TDs. The results highlighted the possibility to detect weaknesses in understanding novel metaphors in children with ACC, in spite of their apparently adequate linguistic capabilities. An early detection of a weakness in this area can pave the way to neurolinguistic treatment in order to enhance the understanding of nonliteral meaning, which, in the developmental trajectory, will be increasingly involved in everyday life communication. Future research should explore more in-depth a capability that intrinsically requires high interconnectivity, such as novel metaphor comprehension, in a brain in development where the major tract connecting the two hemispheres is missing.

## 1. Introduction

Agenesis of the corpus callosum (ACC) is a rare malformation that occurs in 1:4000 live births [1] and results from the failure to develop, either completely or partially, in utero, the largest bundle of fibers connecting cerebral hemispheres. In 30–45% of cases, a specific cause can be identified [2] (10% chromosomic anomalies and 20–35% genetic syndromes). Among the syndromic forms, the best known is Aicardi syndrome, whose genetic origin has been hypothesized but not yet clarified [3]. Most frequently, the etiology is unclear and, in this case, the denomination is isolated ACC. In the general population, individuals with isolated ACC are 1.8 per 10,000 [1] while in children with neurodevelopmental disabilities, for example autism spectrum disorder, these are 230–600 per 10,000 [4]. In the general population, individuals do not have additional syndromes nor other brain pathologies, but their cognitive and behavioral profiles are extremely heterogeneous, as highlighted in a recent meta-analysis [4]. Intellectual development can range from adequate to severely delayed [5]. Neuropsychological profiles of individuals with an intellectual quotient (IQ) above 80 highlight three core aspects (poor interhemispheric sensory-motor integration, slowed cognitive processing, problem with complex reasoning and problem solving). These aspects entail cognitive and social impairments that increase as a function of task complexity [6].

Our focus is precisely on highly demanding linguistic tasks, beyond basic language abilities such as lexical and grammatical production and comprehension, and repetition. By high-level linguistic usages, we refer to those usages where the speaker’s meaning is markedly deviant from the conventional word meaning. The listener must then infer the meaning from the speaker’s communicative intention and the context. The literature reports the finding that adolescents and adults with ACC perform poorly on tasks involving high-level linguistic usages such as idioms, proverbs, and humor [7,8,9]. A more limited number of studies has explored these aspects in children with ACC [10] within their neuropsychological profile, while, to our knowledge, no study has explored novel metaphor comprehension. Novel metaphors differ from conventional or “lexicalized” metaphors inasmuch as they are generated by a spontaneous creative act and, as such, their meaning is not encoded in the repertoire of already known metaphors. As highlighted by Bowdle and Gentner [11], when a metaphor is produced for the first time it is obviously a novel one. Afterwards, when people starts using it, it becomes part of the mental lexicon and its meaning sounds familiar.

The reason for choosing novel metaphors is twofold. The first is related to the neural correlates of novel metaphor comprehension, which requires more activation of brain regions in both hemispheres than literal expressions or conventional metaphors [12,13,14]. It is worthwhile noting that those regions are associated not only to specific linguistic functions but also to theory of mind (e.g., inference of communicative intentions) and executive function (e.g., inhibition). In addition, neuroscientific research has shown that novel and conventional metaphors affect differently the brain mechanisms involved in comprehension [13]. The second reason is developmental, because metaphor comprehension starts to develop at preschool age in typically developing (TD) children when tasks are based on sensory metaphors. A sensory metaphor is so denominated because its constitutive terms both pertain to the physical realm. In the sensory metaphor “The sun is a ball”, both “sun” and “ball” represent objects that are familiar to a child and can be analyzed and explained on verbal grounds by identifying perceptual properties (shape, color, etc.). In addition, this particular metaphor is also a novel one, i.e., unconventional, and can be spontaneously produced by children as young as 4 years. In production, children often assign new words (e.g., “ball”) to familiar things (e.g., “sun”) although they possess the conventional label for these things, and the renaming assumes the form of a metaphor [15,16]. Yet, production and comprehension of sensory metaphors rely on different processes. While, in production, the perception of a similarity between two objects triggers the renaming activity, in comprehension, the contrast between meanings stimulates the recognition of a similarity. Back to the “sun–ball” example, the sentence “the sun is a ball” is literally a false sentence but it becomes “true” if we intend it metaphorically. In development, children increase their ability to inhibit the literal meaning, infer the metaphorical communicative intention, and identify the semantic features shared by the two terms of the metaphor. Several studies, starting from the 1980s, have dealt with these issues also in atypical development, coming to quite heterogeneous results even within the same clinical condition. Different profiles of strong and weak points and possible related factors have been outlined although many issues are still unclear [16,17].

Our study describes novel metaphor comprehension in a child with isolated and complete ACC, who, at his age, should have overcome the early stages of the development of sensory metaphor production–comprehension processes.

## 2. Materials and Methods

### 2.1. Participants

One child (7.2; male) with ACC, conventionally called RJ, recruited at the NESMOS (acronym in English: Neuroscience, Mental Health, Sensory Organs) Department, Faculty of Medicine and Psychology, “Sapienza” University of Rome, and six TD controls participated in this study. At first screening, RJ’s score on the Coloured Progressive Matrices (PM47) [18] was at the 95th percentile, and on Similarities-Wechsler Intelligence Scale IVth Edition (WISC IV) [19], a verbal categorization task, the weighted score was 10 (average).

Six criteria were used to include children in the control group: (a) age range: 7–7.3; (b) gender: male; (c) score range on the PM47: 95th percentile; (d) weighted score range on Similarities: 9–11; (e) no socio-communicative or learning difficulties or neurodevelopmental disorder; (f) socio-cultural background: average; (g) similar school curriculum. The controls were recruited within a project aimed at revalidating a test. The study was approved by the Ethics Committee of the NESMOS Department. Informed consent was given freely by RJ’s parents.

Table 1 reports RJ’s clinical history and current cognitive and linguistic profile. The picture that emerges shows a child essentially adequate on cognitive and linguistic grounds. Considering the existence of comorbidity between ACC and autism spectrum disorder (ASD), as reported by the literature [4,20], we conducted an observation of the behavior of the child and an interview with the parents to identify possible autistic-like features. RJ was reported by his parents to have difficulties in turn-taking in conversation and to remain perplexed when hearing idioms used in familiar contexts and when he had to infer implicit meanings. Beyond these pragmatic difficulties, no other sign suggestive of ASD was found, in particular no excessive focus on restricted and repetitive activities/interests/behaviors, nor sensory abnormalities.

**Figure 1:**
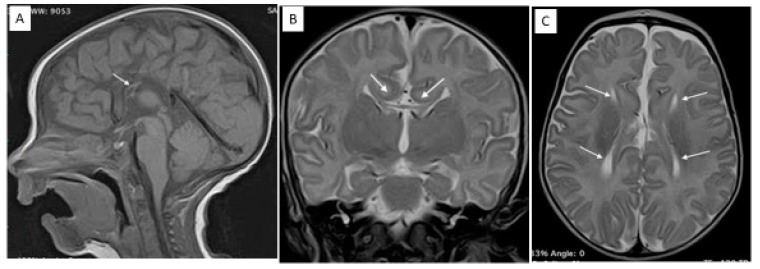
RJ’s magnetic resonance imaging. (**A**) Sagittal T1 WI. The corpus callosum is absent. There is a small residual portion (arrow) in the genu/anterior body. (**B**) Coronal turbo T2 WI. The cingulate gyri are everted as a result of the absence of the callosal fibers (arrows). There is a small residual portion of the genu/anterior body (thin arrows). (**C**) Axial turbo T2 WI. The walls of the lateral ventricles are dysmorphic because the Probst bundles do not cross the midline.

Figure 1 shows RJ’s magnetic resonance imaging at two months.

### 2.2. Instrument and Measures

We used the Junior Metaphor Comprehension Test (Junior MCT) [23], for children 4–6, to assess metaphor comprehension. The test requires participants to explain the meaning of 12 metaphors in decontextualized sentences, and 13 metaphors contextualized in four short stories. The majority of the metaphors are “sensory”, e.g., “The moon is a light bulb”, where “moon” belongs to the semantic area of “celestial bodies” and “light bulb” to that of “electric devices”. This is an example of decontextualized metaphor because it is a sentence without a precise context. An example of contextualized metaphor is the word “nest” in one of the short stories that tells about a little girl who would prefer to remain in her bed/nest instead of getting ready for school.

Responses are coded on a 3-level scale. For reasons of space, we provide examples drawn from the above decontextualized metaphor. Score 0: the child declares he/she just doesn’t know (elusion) or refuses (refusal) the metaphorical use of words (“No, it can’t be”), or interprets the metaphor literally (“Yes, it’s *[the moon]* the light bulb in my room”), or magically (“A magician transformed the moon into a light bulb”) (magical response), or metonymically (“Near the moon, in the sky, there is a bulb”) (metonymical response). Score 1: the child is able to recognize at least one semantic feature shared by the terms of the metaphor, based on functional or perceptual characteristics (“They both give light”; “They are both yellow”). Score 2: both similarities and differences between the two terms of the metaphor are considered (“They both give light but the light bulb, you can switch it on also during the day; the moon is like a slice of an orange but not the light bulb”). Maximum score: 50.

The database of the normative sample was formed by 600 participants from different regions of Italy (4–6 years; *N* = 200 for each year range) with an average socio-cultural background, based on study and professional level of both parents. The validation of the Junior MCT has shown good psychometric characteristics: reliability (Cronbach’s alpha: 0.860), high test-retest correlations (r–tt: 0.848), and high inter-raters’ agreement (0.68 at age 4; 0.73 at age 5; 0.74 at age 6).

Considering RJ’s weakness on Verbal Working Index and Sentence repetition (Table 1), we asked the child to repeat each item twice, once after the examiner’s presentation and another time after his own response. For each item correctly repeated, a score of 1 was attributed. Maximum score: 50.

### 2.3. Procedure

RJ was assessed by two examiners (S.M. and F.B.) in a quiet room of the hospital, without distracting auditory or visual stimuli after a warming-up phase based on conversation, which also aimed at exploring the child’s comprehension and spontaneous speech level.

Considering that the items of the Junior MCT were repeated twice (2.2), the administration of the test was subdivided into two parts, with a short play pause in between. The controls were assessed with the same procedure, in a room of their school by a different examiner (C.P.).

## 3. Results

Before reporting on the performance on the Junior MCT [23], we can note RJ’s excellent performance on item repetition (Table 2). The child could perfectly recall all the 25 items of the test, before and after his responses, as requested.

RJ interpreted two decontextualized and six contextualized metaphors at level 1, all the other items being interpreted at 0 level (Table 2). The total score (8), converted into the T score for 6-year-old children (the normative sample’s age closest to RJ’s), is equivalent to 25, which positions RJ at a deficient level (T ≤ 30), while the controls varied from average to superior. If we compare RJ’s performance to the 5-year-olds of the normative sample, his T score is equivalent to 32 (low-average) (31 < T ≤ 40), and if we compare it to the 4-year-olds, his T score is equivalent to 48 (average) (41 < T ≤ 60). Therefore, RJ’s performance on the Junior MCT positioned him at a 4-year-old child’s level.

Within the score 0, which is predominant (68%), we thought it interesting to see which variant of responses of that level was the most represented and found it was the refusal responses (64.71%), followed by metonymical (23.53%), and literal responses (11.76%).

The controls, as can be seen in Table 2, had T scores that range from average (41–60) to superior (>70).

To compare RJ’s performance to the TDs, the Crawford and Howell’s method [24] was used. This method allows to compare an individual with control groups with modest *N* (e.g., <10). The statistics of the control group are treated as sample statistics and the *t*-distribution (with N − 1 degrees of freedom) is used to evaluate the abnormality of the individual’s scores. The *p*-value refers to the probability for individuals in the population from which the normative sample was drawn to have a score as low as that observed for the individual. All differences were very significant. Decontextualized metaphors: mean: 14.17; SD: 2.27; *t*: −4.964, *p*: 0.002. Contextualized metaphors: mean: 15.67; SD: 2.13; *t*: −4203, *p*: 0.004. Total metaphors: mean: 29.83; SD: 4.06; *t*: −4.978, *p*: 0.004.

## 4. Discussion

RJ’s performance on the Junior MCT [23] highlighted some interesting contrasts. His overall outcome positioned him at a deficient level, with a delay of approximately three years. His performance on linguistic tasks was overall adequate, except for a slight weakness in Sentence completion and a deficient level in Sentence repetition [22].

A deficit in a sentence repetition task may depend on different components, such as phonological discrimination, lexical and grammatical comprehension, among others. However, RJ’s performance on these components in the specific sentence repetition task we administered were all average (Table 1, Current cognitive and linguistic profile). The perfect performance on the Junior MCT item repetition task and the adequate performance on the Word and Non-word repetition task seem to be in contrast with the deficit shown in the Sentence repetition task. However, while the items of the Junior MCT are short sentences and the Word and Non-word repetition task is constituted by single words, the sentences of the Sentence repetition task are quite long and grammatically complex (e.g., “The bedside table, near Francesco’s bed, is bigger than one in Luca’s room”). Most probably, the child’s low outcome in this task can be ascribed to his difficulties in working memory, as attested by his Working Memory Index [19]. We might think that RJ’s difficulties in Sentence completion have influenced the planning of the sentences to respond to the Junior MCT requests. Nevertheless, we must note that the planning required to give level 1 responses, which already reflect a satisfactory access to metaphorical meaning, is not particularly demanding. For instance, for the “moon/light bulb” item, a level 1 response can be a plain sentence such as “they are both yellow”. Such types of responses are also sufficient for the Similarities subtest [19], where RJ’s performance was average.

In the Junior MCT, the majority of the child’s responses (17/25) were at the lowest level (score 0), and, among these, 64.71% were refusals. RJ seemed to deny the legitimacy of the metaphors. His typical reaction was the following: “It’s not true!”, “It can’t be”; “No, never heard…”; “It’s absolutely false”. If we consider another variant of the 0 level responses, namely the literal responses, RJ modified the real world in such a way as to make the sentence of the item literally true, and therefore acceptable to his mind. For instance, in the “bed/nest” metaphor, he said: “The little girl was sleeping in a nest made out of leaves, feathers, grass, and moss… so soft and warm.” In the metonymical responses, another variant of the 0 level responses, the child associated the two terms of the metaphor on the basis of spatial and/or temporal contiguity. For instance, in the item “A cloud is a sponge”, he said: “The sponge flew in the sky over a cloud because the wind was blowing too strongly.”

We might find it surprising that RJ performed so poorly in metaphor comprehension and adequately in Similarities [19]. This contrast could be explained by the fact that, in order to grasp the meaning of a novel metaphor, a consistent inferential effort must be made to compare words belonging to two different semantic categories (“moon” vs. “light bulb”). Although RJ is able to identify some relevant features in the words included in the items of the Similarities subtest, it is to be noted that these words belong to the same semantic category (“butterfly” vs. “bee”). In addition, these words are presented in a pair while the two terms of a metaphor are linked in a sentence by the verb “to be” (“The moon *is* a light bulb”). It is this connection that gives the sentence its unconventional and “false” character if the sentence is taken literally. To face such an unconventional meaning, one must, on the one hand, infer the speaker’s communicative intention, which calls into play theory of mind, and, on the other hand, inhibit those features of the words that are irrelevant to understand the metaphor, which calls into play a typical executive function. Both conditions are needed to fill the gap between what is literally uttered and what is subjectively intended by the speaker. The prevalence of refusals within the 0 level responses could reflect a specific weakness in one or both of these factors.

We believe this study presents some limitations. The first one is intrinsically related to the nature of a case study because the results cannot be generalized. The second limitation is that we have no other measures of RJ’s capabilities likely to be linked to metaphor comprehension on neuropsychological grounds, such as theory of mind or some type of executive function. Lastly, we made no comparison between the results on metaphor comprehension based on explanation, as in the Junior MCT, and those based on a multiple-choice task where the child can recognize a nonliteral meaning without being able to explain it [16].

Our outcomes suggest two major pathways for future studies—one that addresses clinical assessment and intervention and the other that addresses research. From the clinical point of view, our study highlighted that some difficulties in high-level language usages, such as novel metaphor comprehension, could be detected at a very early age in children with isolated ACC, in spite of otherwise adequate cognitive and language capabilities. In addition, identifying such weaknesses in understanding novel metaphors that are already at preschoolers’ reach could offer the opportunity to seize a milestone in the developmental trajectory of this type of comprehension. As this type of capability will be increasingly involved in ever more complex usages in further development, an early detection can pave the way to neurolinguistic treatments in order to enhance high-level language capabilities. Actually, recent studies have described treatments addressing children with ASD where the adult could teach strategies to inhibit the literal meaning of metaphors, train the ability to infer the communicative intention underlying metaphor, and enhance the capability to identify relevant semantic features [25,26,27]. The promising outcomes highlighted by these studies could inspire similar treatments for children like RJ.

From the research point of view, investigating other children with ACC sharing RJ’s characteristics could contribute to identifying a possible subtype within the heterogeneous condition of children with isolated ACC. Future research could explore a capability that intrinsically requires high interconnectivity [12,13,14,28], such as novel metaphor comprehension, in a brain in development where the major tract connecting the two hemispheres is missing.

## 5. Conclusions

Exploring such a rare condition of ACC as the one that characterizes RJ offers a special opportunity to highlight core aspects in the neuropsychological profile of children with ACC. In particular, studying metaphor comprehension (but also metaphor production) since preschool age opens up a window on the intertwined relationship between figurative language, theory of mind and executive function.

## Figures and Tables

**Table 1:** RJ’s clinical history and current cognitive and linguistic profile: phases, data, and assessment tools.

	DATA	
PHASES		ASS. TOOLS
	Born at the 42nd week with eutocic birth. Apgar Index: 8/10, 1st and 5th minute. Absence of perinatal complications. Weight at birth: 4000 gr, length: 55 cm; head circumference: 36.5 cm. Normal pregnancy; no exposure to teratogenic agents nor infections. Negative TORCH screening (Serum testing); fetal echocardiography within the norm. Negative expanded newborn metabolic screening. Family anamnesis reveals no syndromic picture nor nervous system pathology. ACC was identified with morphology ultrasound at the second pregnancy trimester, then confirmed at birth. Brain Computerized Axial Tomography (CAT) performed at 1 month of age and brain Magnetic Resonance Imaging (MRI) at 2 months. No other malformation nor anatomic alteration suggestive of a genetic syndrome appeared at clinical exam. To exclude possible hereditary forms, a genetic study was performed on RJ and his parents, which did not highlight significant alteration (Del Giudice et al., 2020, submitted). No alteration of visual or auditive functions were found. Electroencephalogram (EEG), repeated several times until the age of 7, awake and during sleep, was always within the norm.	
From birth to preschool age	RJ did not follow any pharmacological treatment. Stature and ponderal growth were always within the norm for his age, while motor and language development were slightly delayed (first steps at 18 months and first words at 17 months). Both delays were caught up. The Griffith Mental Development Scales [21], administered at age 3, revealed a relatively homogeneous cognitive profile except a weakness in the visuo-perceptual area, on the basis of which a visuo-spatial treatment was undertaken. An assessment performed at age 4.4 highlighted an IQ of 95, within the norm, and a mental age of 45 months for a chronological age of 48 months.	Griffith Mental Development Scales
	The general intellectual child’s profile was based on the WISC IV [19], from which an IQ of 88 was drawn, that positioned RJ in the average range. This IQ was representative of the child’s intellectual ability, as well as the WISC IV’s indices. Verbal Comprehension Index: 86 (average); Perceptual Reasoning Index: 93 (average); Working Memory Index: 79 (below); Speed Processing Index: 109 (average). The Working Memory Index is a weakness from both the normative and individual points of view.	Wechsler Intelligence Scale IVth Edition (WISC IV)
Current cognitive and linguistic profile	The assessment [22] explored linguistic abilities showing many strengths and one deficient performance. On the production side: denomination within the norm (z: 0); semantic and phonological fluence (z: +1.5), sentence completion (z: -1). On the comprehension side: phonological discrimination, lexical and grammatical comprehension, linguistic and emotional prosody comprehension within the norm (z: 0). Word and non-word repetition: also within the norm (z: 0); Sentence repetition: deficient (z: -2). Speech was good, based on the clinical assessment and parents’ report.	Language Assessment Battery for Children_4-12

**Table 2:** RJ’s scores on metaphor comprehension (Junior Metaphor Comprehension Test (Junior MCT)).

	Rep	Dec Met	Cont Met	Tot Met
	Raw Score	Raw Score	Raw Score	(Raw score) T Score
Child RJ	50	(2)	(6)	(8) T: 25
Control 1	50	(11)	(13)	(24) T: 54
Control 2	50	(13)	(13)	(26) T: 58
Control 3	50	(16)	(17)	(33) T: 71
Control 4	50	(14)	(15)	(29) T:63
Control 5	50	(13)	(18)	(31) T: 67
Control 6	50	(18)	(18)	(36) T: 76

Legend: Rep: repetition of the item. Dec Met: decontextualized metaphors. Cont Met: contextualized metaphors. Tot Met: total metaphor score.
